# Multimodal assessment of mitochondrial function in Parkinson's disease

**DOI:** 10.1093/brain/awad364

**Published:** 2023-12-07

**Authors:** Thomas Payne, Toby Burgess, Stephen Bradley, Sarah Roscoe, Matilde Sassani, Mark J Dunning, Dena Hernandez, Sonja Scholz, Alisdair McNeill, Rosie Taylor, Li Su, Iain Wilkinson, Thomas Jenkins, Heather Mortiboys, Oliver Bandmann

**Affiliations:** Sheffield Institute for Translational Neuroscience, University of Sheffield, Sheffield S10 2HQ, UK; Sheffield Institute for Translational Neuroscience, University of Sheffield, Sheffield S10 2HQ, UK; Sheffield Institute for Translational Neuroscience, University of Sheffield, Sheffield S10 2HQ, UK; Sheffield Institute for Translational Neuroscience, University of Sheffield, Sheffield S10 2HQ, UK; Sheffield Institute for Translational Neuroscience, University of Sheffield, Sheffield S10 2HQ, UK; Institute of Metabolism and Systems Research, College of Medical and Dental Sciences, The University of Birmingham, Birmingham B15 2TT, UK; The Bioinformatics Core, Sheffield Institute of Translational Neuroscience, University of Sheffield, Sheffield S10 2HQ, UK; Molecular Genetics Section, Laboratory of Neurogenetics, NIA, NIH, Bethesda, MD 20814, USA; Neurodegenerative Diseases Research Unit, Laboratory of Neurogenetics, National Institute of Neurological Disorders and Stroke, National Institutes of Health, Bethesda, MD 20814, USA; Department of Neurology, Johns Hopkins University Medical Center, Baltimore, MD 21287, USA; Sheffield Institute for Translational Neuroscience, University of Sheffield, Sheffield S10 2HQ, UK; Statistical Services Unit, The University of Sheffield, Shefield S3 7RH, UK; Sheffield Institute for Translational Neuroscience, University of Sheffield, Sheffield S10 2HQ, UK; Department of Psychiatry, University of Cambridge, Cambridge CB2 0SP, UK; Academic Unit of Radiology, University of Sheffield, Sheffield S10 2JF, UK; Sheffield Institute for Translational Neuroscience, University of Sheffield, Sheffield S10 2HQ, UK; Department of Neurology, Royal Perth Hospital, Perth WA6000, Australia; Sheffield Institute for Translational Neuroscience, University of Sheffield, Sheffield S10 2HQ, UK; Sheffield Institute for Translational Neuroscience, University of Sheffield, Sheffield S10 2HQ, UK; Neuroscience Institute, University of Sheffield, Sheffield S10 2HQ, UK

**Keywords:** Parkinson’s disease, mitochondria, fibroblasts, ^31^phosphorus magnetic resonance spectroscopy, disease stratification

## Abstract

The heterogenous aetiology of Parkinson's disease is increasingly recognized; both mitochondrial and lysosomal dysfunction have been implicated. Powerful, clinically applicable tools are required to enable mechanistic stratification for future precision medicine approaches. The aim of this study was to characterize bioenergetic dysfunction in Parkinson's disease by applying a multimodal approach, combining standardized clinical assessment with midbrain and putaminal 31-phosphorus magnetic resonance spectroscopy (^31^P-MRS) and deep phenotyping of mitochondrial and lysosomal function in peripheral tissue in patients with recent-onset Parkinson's disease and control subjects.

Sixty participants (35 patients with Parkinson's disease and 25 healthy controls) underwent ^31^P-MRS for quantification of energy-rich metabolites [ATP, inorganic phosphate (Pi) and phosphocreatine] in putamen and midbrain. In parallel, skin biopsies were obtained from all research participants to establish fibroblast cell lines for subsequent quantification of total intracellular ATP and mitochondrial membrane potential (MMP) as well as mitochondrial and lysosomal morphology, using high content live cell imaging.

Lower MMP correlated with higher intracellular ATP (*r* = −0.55, *P* = 0.0016), higher mitochondrial counts (*r =* −0.72, *P <* 0.0001) and higher lysosomal counts (*r* = −0.62, *P =* 0.0002) in Parkinson's disease patient-derived fibroblasts only, consistent with impaired mitophagy and mitochondrial uncoupling. ^31^P-MRS-derived posterior putaminal Pi/ATP ratio variance was considerably greater in Parkinson's disease than in healthy controls (F-tests, *P =* 0.0036). Furthermore, elevated ^31^P-MRS-derived putaminal, but not midbrain Pi/ATP ratios (indicative of impaired oxidative phosphorylation) correlated with both greater mitochondrial (*r =* 0.37, *P =* 0.0319) and lysosomal counts (*r =* 0.48, *P =* 0.0044) as well as lower MMP in both short (*r =* −0.52, *P =* 0.0016) and long *(r =* −0.47, *P =* 0.0052) mitochondria in Parkinson's disease. Higher ^31^P-MRS midbrain phosphocreatine correlated with greater risk of rapid disease progression (*r =* 0.47, *P =* 0.0384).

Our data suggest that impaired oxidative phosphorylation in the striatal dopaminergic nerve terminals exceeds mitochondrial dysfunction in the midbrain of patients with early Parkinson's disease. Our data further support the hypothesis of a prominent link between impaired mitophagy and impaired striatal energy homeostasis as a key event in early Parkinson's disease.

## Teaser

Payne *et al.* use ^31^P-MRS to assess brain bioenergetics *in vivo* in patients with Parkinson's disease and combine this with *in vitro* assessment of mitochondrial function in fibroblasts from the same individuals. Markers of impaired brain bioenergetics correlated with several markers of impaired mitophagy in fibroblasts.

## Introduction

Parkinson's disease is the second most common neurodegenerative disorder and increasing in global prevalence.^1^ A key challenge in developing disease-modifying therapies is the inherent clinical and pathogenic heterogeneity in Parkinson's disease. There is therefore a great need to develop tools for the mechanistic stratification in individual patients to develop precision medicine approaches for future clinical trials.^2^

There is strong evidence for mitochondrial and lysosomal dysfunction in Parkinson's disease.^3–6^ Abnormal mitochondrial and lysosomal function and morphology have been demonstrated in the peripheral tissue of both genetic and sporadic Parkinson's disease.^7–9^ Impaired mitophagy has also been implicated in both genetic and sporadic Parkinson's disease.^10,11^ Previously, we undertook deep mechanistic phenotyping of peripheral tissue in a large cohort of sporadic Parkinson's disease and identified distinct subgroups with mitochondrial or lysosomal dysfunction. Mitochondrial dysfunction was rescued in patient tissue with prominent mitochondrial dysfunction, using the putative neuroprotective compound ursodeoxycholic acid (UDCA).^12^ This demonstrates the potential of mechanistic stratification and compound testing in peripheral tissue of individual patients to facilitate precision medicine approaches. However, translation to clinical practice is challenging since obtaining tissue fibroblasts is an invasive procedure, labour-intensive and has conceptual limitations.

^31^Phosphorus magnetic resonance spectroscopy (^31^P-MRS) allows the non-invasive *in vivo* quantification of key bioenergetic metabolites such as adenosine triphosphate (ATP) and phosphocreatine (PCr), enabling the indirect assessment of oxidative phosphorylation.^13^^31^P-MRS may therefore be a suitable non-invasive tool to identify those patients with Parkinson's disease most likely to benefit from putative mitochondrial rescue compounds.

In this study, we undertook multimodal assessment of mitochondrial dysfunction in Parkinson's disease, combining midbrain and putaminal ^31^P-MRS with mechanistic phenotyping in peripheral tissue to comprehensively assess cellular bioenergetics in Parkinson's disease.

We show that mitochondrial and lysosomal function varied markedly in patients with Parkinson's disease and controls. Elevated putaminal inorganic phosphate (Pi)/ATP ratios (reflecting impaired oxidative phosphorylation) correlated strongly with fibroblast-derived indices suggestive of mitophagy in Parkinson's disease only. In contrast, midbrain-derived Pi/ATP ratios did not correlate with mitophagy indicators in peripheral tissue. Our study provides further evidence for early bioenergetic and autophagy impairment at the Parkinson's disease synapse.^14^

We also identified an inverse correlation between midbrain phosphocreatine levels and predicted rapid disease progression in Parkinson's disease, which may facilitate enrichment of future clinical trials for fast versus slow progressors.^15^

## Materials and methods

### Recruitment

Thirty-five participants with recent onset Parkinson's disease (≤3 years since diagnosis) and 25 healthy control subjects with similar age and sex distribution were recruited from movement disorder clinics at Sheffield Teaching Hospitals NHS Foundation Trust and through the Parkinson's UK Research Network. The clinical diagnosis was made by a movement disorders specialist according to the Queen Square Brain Bank Criteria.^16^ Exclusion criteria were contraindications to either MRI or skin biopsy (e.g. through ferromagnetic implants or therapeutic anticoagulation) or evidence of significant cognitive impairment (Mini-Mental State Examination score <25).^17^ All participants provided written informed consent. The study received local Research Ethics Committee approval (REC 18/NW/0328).

### Clinical assessment

A detailed clinical history, neurological examination and assessment with the following clinical rating scales were undertaken: Modified Hoehn and Yahr Staging, Movement Disorders Society-Unified Parkinson's Disease Rating Scale Part 3 (MDS-UPDRS-III) and Movement Disorders Society Non-Motor Symptom Scale (MDS-NMSS).^18–21^

A predictive disease progression score was calculated in all patients with Parkinson's disease based on a validated prognostic model, which estimates the risk of an unfavourable outcome as defined by the presence of either postural instability or dementia at 5 years.^15^

### Genetic analysis

All participants supplied an EDTA blood sample for genetic analysis to known pathogenic mutations in monogenic Parkinson's disease genes (e.g. *PINK1*, *PARK2*, *LRRK2*) and any variants of *GBA1* associated with increased risk of Parkinson's disease using the NeuroChip platform.^22^ All detected variants were searched in dbSNP (https://www.ncbi.nlm.nih.gov/snp/) and then classified according to published guidelines.^23^ As any statistical comparisons between genotypes is not feasible with these numbers, data presented do not differentiate the genotypes of Parkinson's disease.

### Fibroblast cell line assessment

Skin biopsies were obtained using a 3 mm punch biopsy needle from the upper forearm and fibroblast lines were established according to previously reported protocols.^24^ Assays were performed under two conditions; glucose containing media (1000 mg/l) or glucose-free media containing 5 mM galactose. Two participants were unable to have fibroblast cell lines established due to COVID-19 restrictions impacting research practice at the time of recruitment. One further control fibroblast cell line was excluded from analysis due to recurrent cell line infections. Therefore, a total of 57 cell lines (Parkinson's disease *n* = 34, controls *n* = 23) were available for analysis.

Mitochondrial membrane potential (MMP) and total intracellular ATP levels were assessed as previously described.^7^ Mitochondrial and lysosomal morphology was quantified by staining live fibroblasts with 80 nM tetramethlyrhodamine (TMRM), 1 µM LysoTracker® (Life Technologies) and 1 µM Hoescht for 1 h prior to imaging, using either the InCell Analyzer 2000 high-content imager (GE Healthcare) or Opera Phenix (Perkin Elmer) imaging systems, as previously described.^25^

Raw images were segmented, analysed and parameters quantified using the InCell Developer software (GE Healthcare) or Harmony software (Perkin Elmer). A custom imaging protocol was used for the segmentation of nuclei, lysosomes and mitochondria to generate total counts for each and morphology parameters, similar to custom imaging protocols previously published.^12^ The mitochondrial population was further subdivided into long and short mitochondria for the assessment of MMP in these subpopulations using a form factor measurement defined as (Pm^2^) / (4πAm), where *Pm* is the length of mitochondrial outline and *Am* is the area of mitochondrion. Short mitochondria are more likely to be destined for mitophagy; this is particularly true in shorter mitochondria with low MMP.^26^ Analysis outputs were consistent across imagers and software, enabling combination of groups in the final analysis.

### ^31^P magnetic resonance spectroscopy

#### Acquisition

^31^P-MRS scans were obtained using a Philips Ingenia 3 T system (Philips Healthcare) and a transmit-receive dual-tuned ^1^H/^31^P birdcage quadrature head-coil (Rapid Biomedical). Two-dimensional chemical shift imaging (CSI) with image-selected *in vivo* spectroscopy was used for spectral spatial localization. Two separate CSI sequences were obtained focused on the midbrain and the putamen. Acquisition parameters for the midbrain CSI were: repetition time = 4000 ms, echo time = 0.22 ms, number of signal averages = 8, sampling points = 2048, spectral bandwidth = 3000 Hz, flip angle = 90°, slice thickness = 20 mm, field of view = 210 mm^2^, acquired voxel sizes of 40 × 40 × 20 mm^3^ and, following k-space filtering and zero filling using a reconstruction matrix of 14 × 14, reconstructed voxel size = 15 × 15 × 20 mm^3^. The putamen CSI acquisition parameters differed only in number of signal averages = 10 and reconstructed voxel size = 17.5 × 17.5 × 20 mm^3^ using a reconstruction matrix of 12 × 12. All CSI sequences used adiabatic pulses, with a second order pencil-beam shim and WALTZ-4 broadband heteronuclear decoupling with nuclear Overhauser effect.

**Figure 1:**
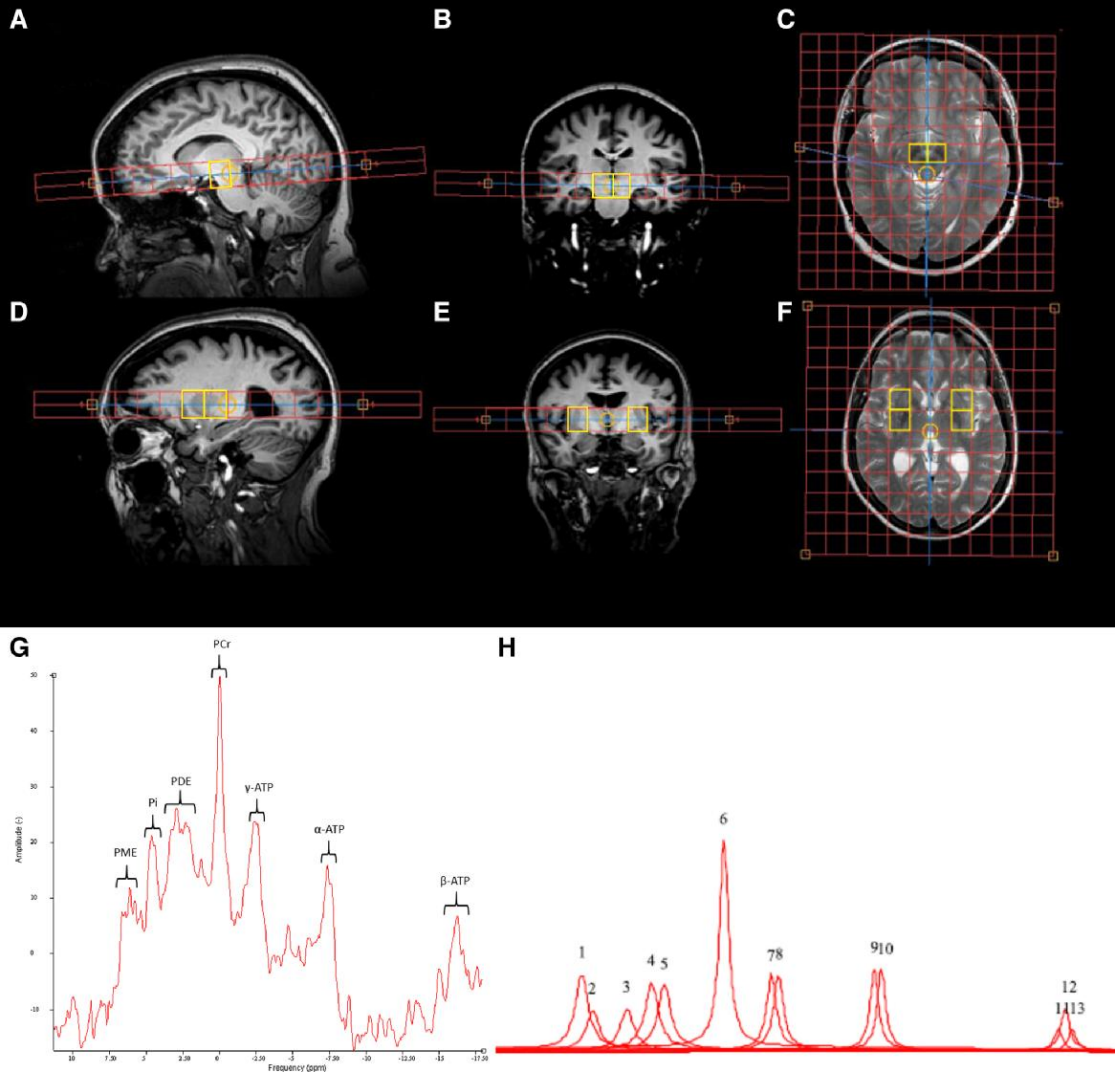
**^31^P-MRS allows quantification of energy-rich metabolites *in vivo***. (**A**) Sagittal, (**B**) coronal and (**C**) axial images demonstrating spectroscopic grid (14 × 14) positioning for the midbrain voxels. Analysed voxels are highlighted in yellow and placement is performed to capture a voxel from the right and left side. Analysis focused on the mean values of the right and left voxels for each of the midbrain, posterior putamen and anterior putamen voxels. Voxel placement ensures the substantia nigra will be included within the voxel of interest. (**D**) Sagittal, (**E**) coronal and (**F**) axial images demonstrating spectroscopic grid (12 × 12) positioning for the putaminal voxels. Analysed voxels are highlighted in yellow and placement is performed to capture a voxel from the right and left side for both the anterior and posterior putamen. (**G**) An example spectrum obtained from the midbrain of a healthy control. This spectrum has been phased and apodized to aid visualization with phosphocreatine frequency shifted to 0 ppm, for quantification spectra are not apodized. (**H**) An example output of peak fitting following AMARES analysis. Thirteen resonances are fitted: 1 = phosphocholine; 2 = phosphoethanolamine; 3 = Pi (inorganic phosphate); 4 = glycerophopshocholine; 5 = glycerophosphoethanolamine; 6 = phosphocreatine; 7 and 8 = γ-ATP; 9 and 10 = α-ATP; 11–13 = β-ATP. Phosphocholine and phosphoethanolamine form the phosphomonoesters (PME). Glycerophosphocholine and glycerophosphoethanolamine form the phopshodiesters (PDE). PME and PDE are only quantified to facilitate normalization of each amplitude to the total phosphorus signal detected in the spectra and were not used in any statistical analysis.

Alignment of CSI sequences was guided by a T_2_-weighted spin-echo image: repetition time = 3000 ms, echo time = 80 ms, flip angle = 90°, slice thickness = 4 mm, field of view = 230 mm^2^, acquisition voxel 0.55 × 0.65 × 4.0 mm^3^, reconstruction matrix = 432 × 432 mm^2^, reconstructed voxel size = 0.53 × 0.53 × 4.0 mm^3^. A 3D T_1_ inversion-recovery volumetric image was obtained and aligned to each CSI acquisition to allow co-localization and correction for partial volume effects: repetition time = 8.3 ms, echo time = 3.8 ms, field of view = 240 mm^2^, flip angle = 8°, inversion time = 1000 ms, slice thickness = 1 mm, acquired voxel size = 1.2 × 1.2 × 1.5 mm, reconstruction matrix = 256 × 256 mm^2^, reconstructed voxel size = 0.94 × 0.94 × 1 mm^3^. Details of spectral localization and voxels of interest are shown in Fig. 1.

#### Spectroscopic data processing

All spectroscopic data were anonymized at acquisition and analysed blinded to participant status. Spectra were processed in the time domain using jMRUI software V5.2 (http://www.jmrui.eu) and signal fitting was performed using the advanced method for accurate, robust and efficient spectral fitting (AMARES) algorithm to determine the relative area under each peak.^27,28^ In each participant, six voxels were selected representing left midbrain, right midbrain, left posterior putamen, right posterior putamen, left anterior putamen and right anterior putamen for subsequent analysis.

Manual preprocessing included zero and first-order phasing for purely absorptive line shapes with no apodization performed. All spectra were frequency shifted to 0 parts per million for phosphocreatine.

A total of 13 resonances were fitted with assumed Lorentzian line shapes, with γ-ATP and α-ATP having doublet peaks with amplitudes and line-widths constrained to a 1:1 ratio to each other and β-ATP having triplet peaks with amplitudes constrained in a ratio of 0.5:1:0.5 and line-widths constrained to a ratio of 1:1:1. Coupling constants for ATP multiplets were set at 18 Hz, as used in previous literature and soft constraints limited ATP line-widths to 5–35 Hz.^29,30^ Additional soft constraints were used to control line-widths for phosphocreatine (5–20 Hz) and all other remaining resonances to 5–30 Hz.^31,32^ All amplitudes were normalized to the total phosphorus signal detected within the respective voxel prior to any statistical analyses. The three multiplets of ATP were summed to calculate total ATP. All spectra were visually inspected for quality of fit and spurious signals were excluded, according to recently published consensus criteria.^33^ Example spectra pre- and post-analysis are shown in Fig. 1. In addition to reporting normalized amplitudes of phosphocreatine, inorganic phosphate and ATP normalized to total phosphorus, a further analysis was performed focusing on the ratio of the raw amplitudes of Pi/ATP in each participant.

T_1_-weighted images were segmented using Statistical Parametric Mapping software (SPM12, https://www.fil.ion.ucl.ac.uk/spm/software/spm12/) and co-registered to the voxel of interest using Gannett (http://www.gabamrs.com/) and MATLAB and Statistics Toolbox Release R2020a (The MathWorks, Inc., Natick, Massachusetts, USA). Each MRS voxel was segmented into grey matter, white matter and CSF. To represent partial volume effects in the midbrain the brain proportion (grey matter plus white matter divided by total voxel volume) was calculated for midbrain voxels, there is a small proportion of segmented grey matter in the midbrain and therefore CSF is most likely to affect MRS signal in these MRS voxels. To represent partial volume effects in the putamen, the grey matter proportion was calculated, given essentially no CSF in these voxels the proportion of grey matter is most likely to influence MRS signal as observed in quantitative proton MRS.^33,34^

Sixty ^31^P-MRS datasets were obtained from the putamen and 59 from the midbrain. Midbrain ^31^P-MRS values from one control were excluded from further analysis due to a spuriously low, non-physiological phosphocreatine level.

### Statistical analysis

Primary ^31^P-MRS parameters of interest were determined *a priori* as total levels of ATP, Pi and phosphocreatine as the mean of the midbrain voxels, the posterior putamen voxels and the anterior putamen voxels, normalized to total phosphorus. To detect group differences, ^31^P-MRS parameters of interest were entered as the response variable into a linear regression model, with the predictor variables specified as disease group, age and sex. This decision was based on previous work, which showed differences between sexes in Parkinson's disease with respect to ATP and phosphocreatine measurements and changes in pH and phosphocreatine with age in the healthy brain.^35,36^^31^P-MRS parameters of interest and partial volume measures were assessed graphically and by correlation analysis. There were no significant correlations between ^31^P-MRS parameters and partial volume measures; therefore, partial volume was not included in subsequent linear regression models to limit the number of covariates respective to sample size (data not shown).

All data for each fibroblast line were normalized to the controls on each plate. Key primary fibroblast parameters of interest specified *a priori* were intracellular ATP, MMP (in all mitochondria, long mitochondria only and short mitochondria only), mitochondrial count per cell and lysosome count. Group differences were assessed using *t*-tests.

All primary ^31^P-MRS metabolite concentrations and fibroblast-derived parameters were normally distributed in healthy controls, as assessed with the visual inspection of Q-Q plots. In both Parkinson's disease and healthy control groups, there were no significant correlations between either age or sex with ^31^P-MRS metabolite levels or fibroblast assay parameters of interest (data not shown).

Differences in variances for all ^31^P-MRS and fibroblast assay parameters between groups were analysed using the F-test of equality of variances. Graphical displays and Pearson's correlation coefficient was used to assess relationships between each ^31^P-MRS parameter and continuous clinical variables of interest. The same analysis was also undertaken to investigate correlations between all fibroblast-derived parameters with each other. For both of these analyses, all *P*-values were adjusted for multiple comparisons using the Benjamini-Hochberg method.^37^

To assess associations between ^31^P-MRS and fibroblast data in a common domain, all relevant ^31^P-MRS parameters and each individual biological fibroblast assay repeat were first transformed to *z*-scores. First each parameter in the healthy control data was transformed to a *z*-score with mean of 0 and standard deviation (SD) of 1. *Z*-scores for patients were then calculated with reference to healthy control population distribution.

For fibroblast data, and for each media condition, each biological repeat was combined into a composite *z*-score with equal weighting given to each individual repeat using the below equation.^38^


$$z_{xc}=\;\frac{\sum_{i=0}^{n}\;z_{x}}{\sqrt{n+2r_{sum}}}$$

where $z_{xc}$ is the composite score, $z_{x}$ is the *z*-scored component and $r_{sum}$ is the sum of the Pearson correlation coefficients between each and every individual *n* components.

Each media condition *z*-score was then combined using the same method to generate a single composite *z*-score for each fibroblast assay parameter that summarizes data from both media conditions. Composite *z*-scores were also generated using the same above method to reflect ^31^P-MRS parameters as either the composite of all four putaminal voxels or a composite of both midbrain voxels. The further exploratory analysis of composite *z*-scored putaminal Pi/ATP ratio was also calculated using this method.

Pearson's correlation coefficient was used to assess relationships between composite *z*-scores derived from ^31^P-MRS and composite *z*-scores of fibroblast assay parameters within each disease group. Given the exploratory nature of these relationships, these correlation analyses were not corrected for multiple comparisons. Any significant correlations identified in either group between ^31^P-MRS and fibroblast assay parameters were further assessed in a sensitivity analysis with linear regression with the additional covariates of age and sex entered to confirm whether the relationship remained significant. These sensitivity analyses were only reported if the observed relationship lost significance. Correlations were further compared between groups using Fisher's *z*-transformation to investigate if correlations between ^31^P-MRS and fibroblast assay parameters differed significantly between groups.

All statistical analyses were completed in R version 4.1.0.

## Results

Demographic characteristics are summarized in Table 1. Groups were well matched, with no significant differences between age, sex or family history of Parkinson's disease between patients and controls. There were two cases of familial Parkinson's disease, a 50-year-old female homozygous for a parkin (*PRKN*) mutation (c.337-376del) and a 28-year-old female heterozygous for the G51D *SNCA* mutation.^39,40^ No other *PARK* gene mutations or *GBA* Parkinson's disease risk variants were detected in the Parkinson's disease patients’ cohort. Neither pathogenic mutations in *PARK* genes nor *GBA* Parkinson's disease risk variants were detected in the control cohort.

**Table 1:** Demographic and clinical features of the study cohort

	Control (*n* = 25)	Parkinson's disease (*n* = 35)	*P*-value
**Sex**			
Male, *n* (%)	12 (48)	19 (54)	0.8272^a^
Female, *n* (%)	13 (52)	16 (46)	
**Age**			
Minimum	28	28	0.839^b^
Maximum	81	82	
Mean ± SD	60.64 ± 10.96	60.06 ± 10.74	
**Family history of Parkinson’s disase in a first-degree relative**			
Yes, *n* (%)	6 (24)	4 (11)	0.535^a^
No, *n* (%)	19 (76)	31 (89)	
**Disease duration (months)**			
Minimum	NA	2	
Maximum	NA	32	
Mean ± SD	NA	13.71 ± 7.50	
**Modified Hoehn and Yahr**			
Minimum	NA	1	
Maximum	NA	3	
Mean ± SD	NA	2.01 ± 0.39	
**MDS-UPDRS Part III scores**			
Minimum	NA	14	
Maximum	NA	54	
Mean ± SD	NA	32.60 ± 9.89	
**Non-Motor Symptom Scale**			
Minimum	NA	3	
Maximum	NA	119	
Mean ± SD	NA	40.09 ± 31.99	
**Total Levodopa equivalent daily dosage (mg)**			
Minimum	NA	0	
Maximum	NA	1000	
Mean ± SD	NA	370.43 ± 218.44	
**Predictive Risk Score**			
Minimum	NA	0.03	
Maximum	NA	0.91	
Mean ± SD	NA	0.40 ± 0.25	

MDS-UPDRS = Movement Disorders Society-Unified Parkinson's Disease Rating Scale; NA = not applicable; SD = standard deviation.

^a^Chi-squared test.

^b^Independent samples *t*-test with Welch's correction.

### Mitochondrial and lysosomal function differs markedly in fibroblasts of patients with Parkinson's disease and controls

**Figure 2:**
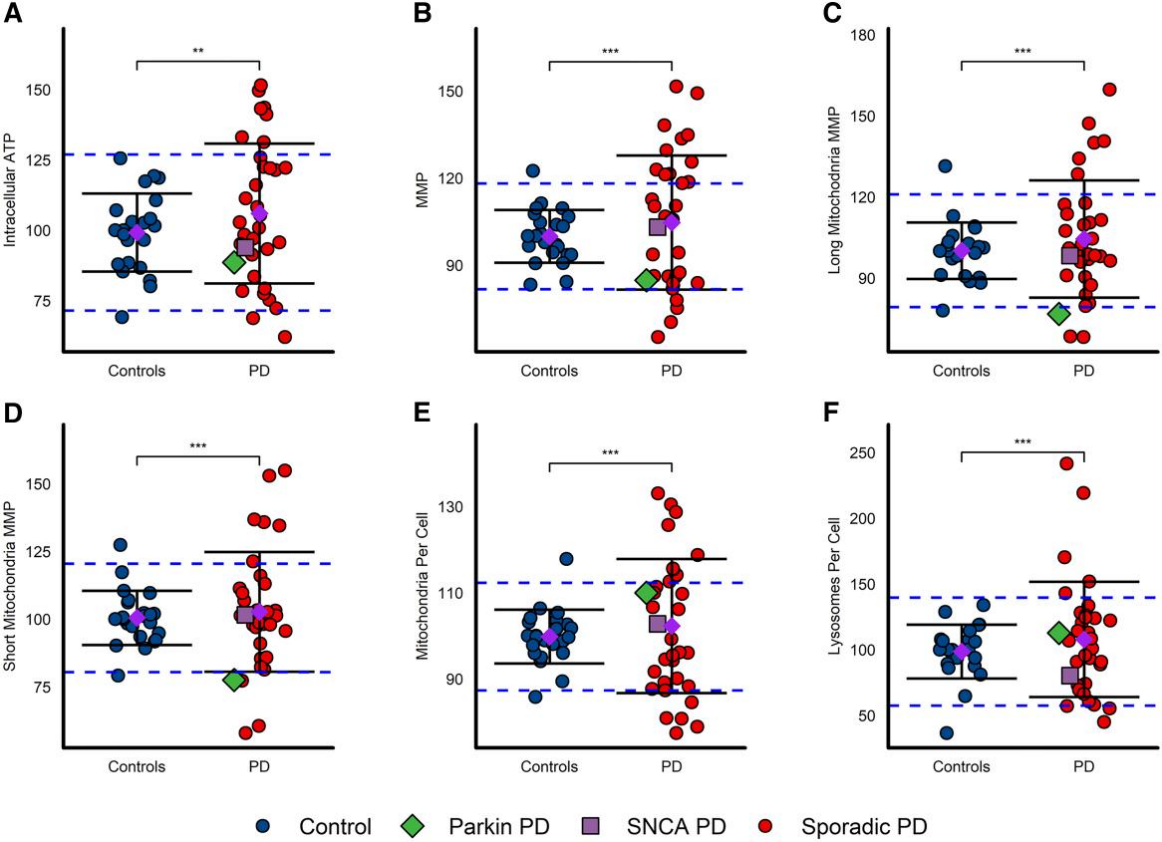
**Considerably greater variance of mitochondrial and lysosomal function in fibroblasts derived from patient with Parkinson's disease**. (**A**) Intracellular ATP, *F*-test *P* = 0.0056; (**B**) MMP, *F*-test *P* < 0.0001; (**C**) long mitochondria MMP only, *F*-test *P* = 0.0007; (**D**) short mitochondria MMP, *F*-test *P* = 0.0003; (**E**) mitochondria count per cell, *F*-test *P* < 0.0001; (**F**) lysosomal count per cell, *F*-test *P* < 0.0001. Mean (purple diamond) ± standard deviation (SD) presented. Blue dashed lines denote 2 SDs from the control mean values. The parkin (*PRKN*^−/−^) mutant patient is depicted with a green diamond, the *SNCA-G51D* mutant patient is depicted with a purple square. All fibroblast assays repeated in triplicate. Data presented are from experiments performed in glucose-containing media and all data from each participant are normalized to mean control values per repeat. Variances between groups were tested with the *F*-test of equality of variances and statistical significance is displayed on each panel. Control: *n* = 23, Parkinson's disease: *n* = 34. *P < 0.05, **P < 0.01, ***P < 0.001. MMP = mitochondrial membrane potential; PD = Parkinson’s disease.

Fibroblast assay results performed in glucose-containing media are summarized in Table 2. Seven of 34 patients had intracellular ATP values outside 2 SDs from the healthy control mean (*F*-test *P* = 0.0056) (Fig. 2A). Fourteen of 34 patients had MMP values outside 2 SDs of the healthy control mean (*F*-test *P* < 0.0001) (Fig. 2B); this was the case for both the MMP in long (8/34 patients, *F*-test *P* = 0.0007; Fig. 2C) and short mitochondria (9/34 patients, *F*-test *P* = 0.0003; Fig. 2D). Fifteen of 34 patients had mitochondria counts per cell outside 2 SDs (*F*-test *P* < 0.0001; Fig. 2E) and 6/34 patients had lysosomal counts outside 2 SDs (*F*-test *P* < 0.0001, Fig. 2F). Overall, the results of all parameters were similar between glucose and galactose media conditions, hence the combined *z*-score accounting for data in both media types was used for further analysis (Supplementary Table 1 and Supplementary Fig. 1).

**Table 2:** Fibroblast assay summary results

Fibroblast assay parameters	Control (*n* = 23)	Parkinson's disease (*n* = 34)	*F*-test	T-test
Intracellular ATP	99.164 ± 13.864	105.928 ± 24.845	*P* = 0.0056	*P* = 0.1946
MMP	99.902 ± 9.114	104.64 ± 23.127	*P* < 0.0001	*P* = 0.2869
Short mitochondria MMP	100.458 ± 10.019	102.678 ± 22.102	*P* = 0.0003	*P* = 0.6102
Long mitochondria MMP	100.108 ± 10.438	104.444 ± 21.702	*P* = 0.0007	*P* = 0.3194
Mitochondria per cell	99.810 ± 6.236	102.268 ± 15.571	*P* < 0.0001	*P* = 0.4120
Lysosomes per cell	98.505 ± 20.522	112.592 ± 51.366	*P* < 0.0001	*P* = 0.1570

Mean and standard deviation and *F*-test shown for all fibroblast data in glucose-containing media. All values displayed are normalized to the mean control values obtained in these conditions. Group differences in fibroblast parameters assessed with *t*-tests. MMP = mitochondrial membrane potential.

We observed that control fibroblasts and patient fibroblast lines with low MMP in glucose remained largely similar across glucose and galactose media. However, in patient lines with a high MMP in glucose we observed the MMP to greatly reduce in galactose. Owing to the bidirectional nature of the data and proportion of patient lines falling into these categories, further formal statistical testing of this observation was not appropriate (data not shown).

**Figure 3:**
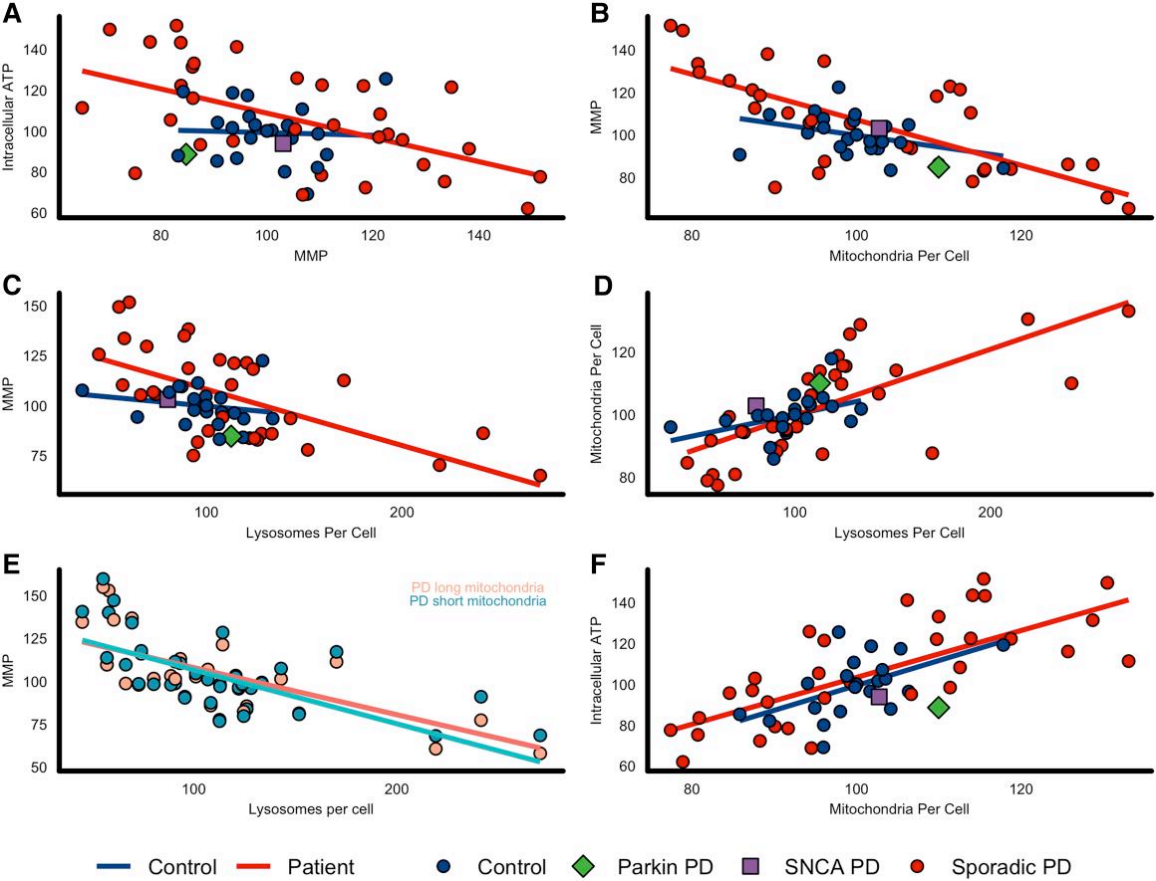
**Analysis of mitochondrial and lysosomal function suggests impaired mitophagy and uncoupling in Parkinson's disease patient fibroblasts**. Fibroblast measures of mitochondrial and lysosome function/morphology; correlations between measures for both Parkinson's disease and controls assess using Pearson's correlation coefficient. (**A**) Intracellular ATP and MMP (control *r* = −0.044, *P* = 0.918; Parkinson's disease *r* = −0.552, *P* = 0.0018); (**B**) MMP and mitochondria per cell (control *r* = −0.379, *P* = 0.0895; Parkinson's disease *r* = −0.723, *P* < 0.0001); (**C**) MMP and lysosomes per cell (control *r* = −0.205, *P* = 0.432; Parkinson's disease *r* = −0.623, *P* = 0.0003); (**D**) mitochondria per cell and lysosomes per cell (control *r* = 0.43, *P* = 0.0603; Parkinson's disease *r* = 0.7, *P* < 0.0001). (**E**) Long mitochondria MMP (peach) and short mitochondria MMP (turquoise) and lysosomes per cell in patients only (Parkinson's disease long mitochondria MMP *r* = −0.65, *P* < 0.0001; Parkinson's disease short mitochondria MMP *r* = −0.73, *P* < 0.0001). (**F**) Intracellular ATP and mitochondria per cell (control *r* = 0.548, *P* = 0.0137; Parkinson's disease *r* = 0.719, *P* < 0.0001). All fibroblast assays repeated in triplicate. Data presented are from experiments performed in glucose-containing media and all data from each participant are normalized to mean control values per repeat. The parkin (*PRKN*^−/−^) mutant patient is depicted with a green diamond, the *SNCA-G51D* mutant patient is depicted with a purple square. Control (blue) *n* = 23, Parkinson's disease (red) *n* = 34. The *P-*values reported are adjusted for multiple comparisons using the Benjamini-Hochberg method. *P < 0.05, **P < 0.01, ***P < 0.001. MMP = mitochondrial membrane potential; PD = Parkinson’s disease.

MMP inversely correlated with both intracellular ATP (*r* = −0.552, *P* = 0.0018) (Fig. 3A) and mitochondrial counts (*r* = −0.723, *P* < 0.0001, Fig. 3B), in Parkinson's disease, but not in controls (*r* = −0.044, *P* = 0.918 and *r* = −0.379, *P* = 0.0895, respectively). Lysosomal counts correlated inversely with MMP in Parkinson's disease only (*r* = −0.623, *P* = 0.0003, Fig. 3C) but not in controls (*r* = −0.205, *P* = 0.432). Lysosomal counts directly correlated with mitochondrial counts in Parkinson's disease only (*r* = 0.7, *P* < 0.0001, Fig. 3D), but not in controls (*r* = 0.43, *P* = 0.0603). In Parkinson's disease, this correlation was similar but marginally larger in short mitochondria (*r* = −0.73, *P* < 0.0001) than long mitochondria (*r =* −0.65, *P* < 0.001, Fig. 3E)

In Parkinson's disease, the association of lower MMP with a higher count for both mitochondria and lysosome would be in keeping with fragmentation of mitochondria with low MMP, the first step in the induction of mitophagy.^41,42^ Intracellular ATP directly correlated with mitochondrial counts in both Parkinson's disease (*r* = 0.719, *P* < 0.0001) and healthy controls (*r* = 0.548, *P* = 0.0137, Fig. 3F). Given the relationships described above between low MMP and high ATP in Parkinson's disease only, the mechanisms underlying the correlation of high ATP and high mitochondrial count may be fundamentally different in Parkinson's disease compared to healthy controls. The paradoxical rise in ATP with low MMP and high mitochondrial counts in Parkinson's disease patient cells may reflect mitochondrial biogenesis in response to mitochondrial uncoupling, as previously observed in other models or activation of other metabolic pathways to compensate for dysfunctional mitochondria.^43^ In healthy controls the correlation between higher ATP and higher mitochondrial counts may simply reflect a greater overall functioning mitochondrial network, which is supported by the lack of correlations seen in controls between MMP and the other parameters to indicate any dysfunction otherwise.

Overall, these data support the hypothesis that the initiation step in sensing dysfunctional mitochondria and subsequent fragmentation of the network is intact in Parkinson's disease, hence the higher number of mitochondria with low MMP. However, the next steps in the process of effectively targeting those mitochondria to be degraded via engulfment in the autophagosome and ultimate fusion with lysosome are not functioning effectively, therefore the dysfunctional mitochondria are not recycled and accumulate within the cell.

### Putaminal Pi/ATP ratios correlate with lower MMP and higher lysosome counts in Parkinson's disease

Degeneration of the dopaminergic nigrostriatal axon terminals is more pronounced than degeneration of the nigral dopaminergic neurons in early Parkinson's disease and there is strong experimental evidence for early impairment of both bioenergetics and autophagy at the Parkinson's disease synapse.^14,44–47^ We therefore hypothesized that impaired oxidative phosphorylation in the putamen (as reflected by elevated ^31^P-MRS-derived Pi/ATP ratios) would correlate more closely with peripheral indices of impaired mitochondrial function and autophagy than in the midbrain in our early Parkinson's disease cohort.^13^

**Figure 4:**
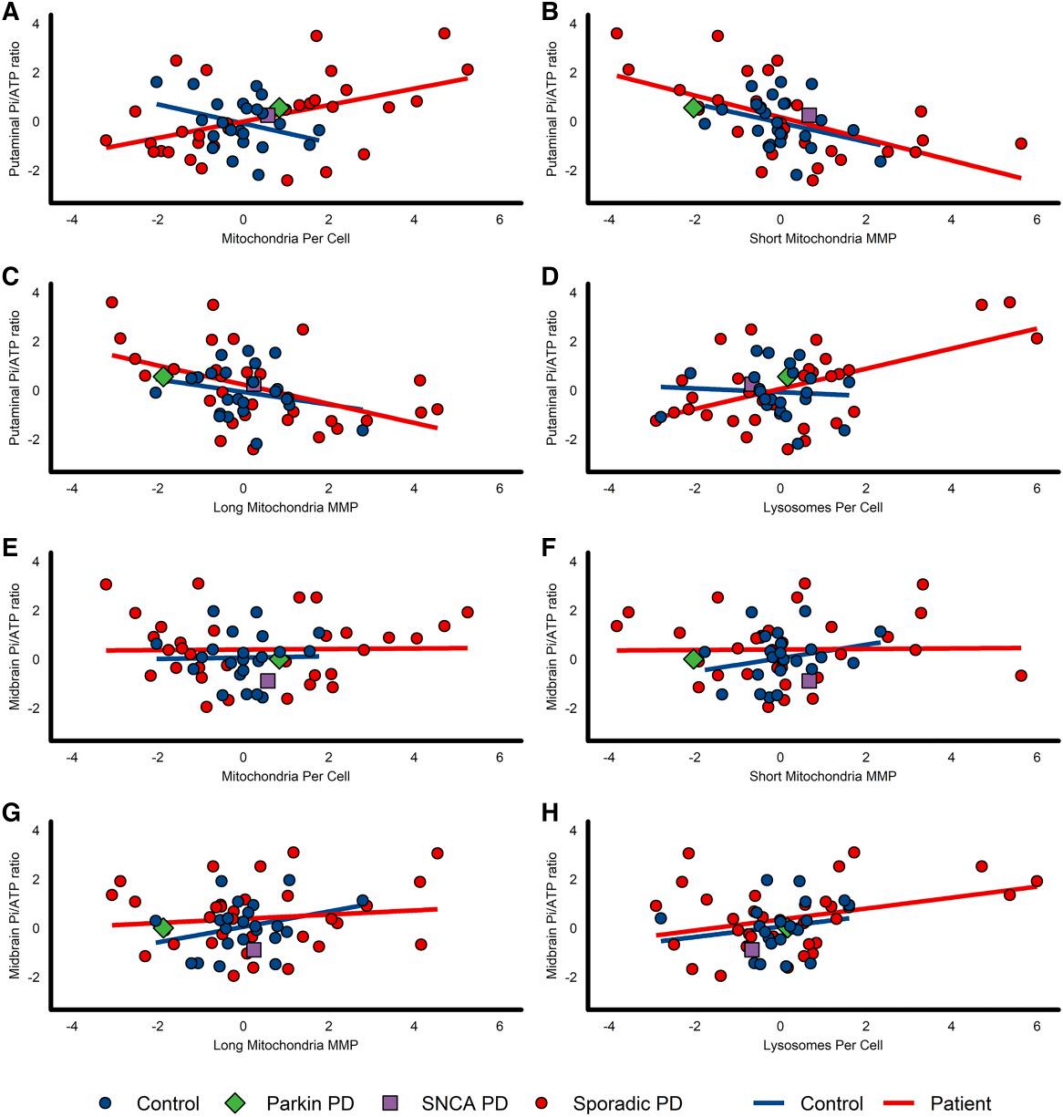
**Putaminal, but not midbrain Pi/ATP ratios correlate with indices of mitophagy in Parkinson's disease patient-derived fibroblasts**. All ^31^P-MRS data are expressed as composite *z*-scored data of all putaminal voxels. All fibroblast assay data are expressed as a composite *z*-score of triplicate repeats in both glucose-containing and galactose-containing media. Pearson's correlation coefficient was used for all analyses. The parkin (*PRKN*^−/−^) mutant patient is depicted with a green diamond, the *SNCA-G51D* mutant patient is depicted with a purple square. (**A**) Putaminal Pi/ATP ratio and mitochondria per cell (control *r* = −0.326, *P* = 0.13; Parkinson's disease *r* = 0.369, *P* = 0.0319). (**B**) Putaminal Pi/ATP ratio and MMP in short mitochondria only (control *r* = −0.345, *P* = 0.107; Parkinson's disease *r* = −0.522, *P* = 0.0016). (**C**) Putaminal Pi/ATP ratio and MMP in long mitochondria only (control *r* = −0.249, *P* = 0.252; Parkinson's disease *r* = −0.469, *P* = 0.0052). (**D**) Putaminal Pi/ATP ratio and lysosomes per cell (control *r* = −0.073, *P* = 0.741; Parkinson's disease *r* = 0.476, *P* = 0.0044). (**E**) Midbrain Pi/ATP ratio and mitochondria per cell (control *r* = 0.023, *P* = 0.92; Parkinson's disease *r* = 0.018, *P* = 0.919). (**F**) Midbrain Pi/ATP ratio and MMP in short mitochondria only (controls *r* = 0.245, *P* = 0.271; Parkinson's disease *r* = 0.016, *P* = 0.93). (**G**) Midbrain Pi/ATP ratio and MMP in long mitochondria only (controls *r* = 0.308, *P* = 0.164; Parkinson's disease *r* = 0.124, *P* = 0.484). (**H**) Midbrain Pi/ATP ratio and lysosomes per cell (controls *r* = 0.183, *P* = 0.414; Parkinson's disease *r* = 0.345, *P* = 0.0457). Putamen data: controls *n* = 23, Parkinson's disease *n* = 34. Midbrain data: controls *n* = 22, Parkinson's disease *n* = 34. MMP = mitochondrial membrane potential; PD = Parkinson’s disease.

As predicted, ^31^P-MRS-derived putaminal Pi/ATP ratios directly correlated with fibroblast-derived mitochondrial counts in Parkinson's disease only, but not in controls (*r* = 0.369, *P* = 0.0319 and *r* = −0.326, *P* = 0.13, respectively) (Fig. 4A). ^31^P-MRS-derived putaminal Pi/ATP ratios were inversely correlated with MMP in both short and long mitochondria in Parkinson's disease patient tissue, but not in controls (short mitochondria: patients *r* = −0.522, *P* = 0.0016; controls *r* = −0.345, *P* = 0.107, Fig. 4B; long mitochondria: patients *r* = −0.469, *P* = 0.0052; controls *r* = −0.249, *P* = 0.252, Fig. 4C). Furthermore, ^31^P-MRS-derived putaminal Pi/ATP ratios directly correlated with lysosome counts in Parkinson's disease (*r* = 0.476, *P* = 0.0044, Fig. 4D), but not in controls (*r* = −0.073, *P* = 0.741). The specific association of elevated ^31^P-MRS-derived putaminal (rather than midbrain) Pi/ATP ratios with reduced MMP and greater lysosome counts in peripheral tissue would be in keeping with prominent abnormal oxidative phosphorylation and impaired mitophagy at the Parkinson's disease synapse.

Individual correlation analysis for both ^31^P-MRS-derived putaminal ATP and Pi were also consistent with the above data, further supporting the assumption that the increased Pi levels were likely to result from increased ATP hydrolysis or impaired ATP synthesis rather than due to a different mechanism (Supplementary Fig. 2).

In contrast, ^31^P-MRS-derived midbrain Pi/ATP ratios did not show any correlations with either mitochondrial counts (patients *r* = 0.018, *P* = 0.919; controls *r* = 0.023, *P* = 0.920, Fig. 4E), short mitochondria MMP (patients *r* = 0.016, *P* = 0.930; controls *r* = 0.245, *P* = 0.271, Fig. 4F) or long mitochondria MMP (patients *r* = 0.124, *P* = 0.484; controls *r* = 0.308, *P* = 0.164, Fig. 4G) in either group with the exception of Pi/ATP ratios directly correlating with lysosome counts in Parkinson's disease only (patients *r* = 0.345, *P* = 0.0457; controls *r* = 0.183, *P* = 0.414, Fig. 4H).

There were no significant correlations between ^31^P-MRS-derived putaminal phosphocreatine and any fibroblast-derived measure, nor between ^31^P-MRS-derived midbrain ATP, phosphocreatine and Pi with any fibroblast-derived parameters (data not shown).

**Figure 5:**
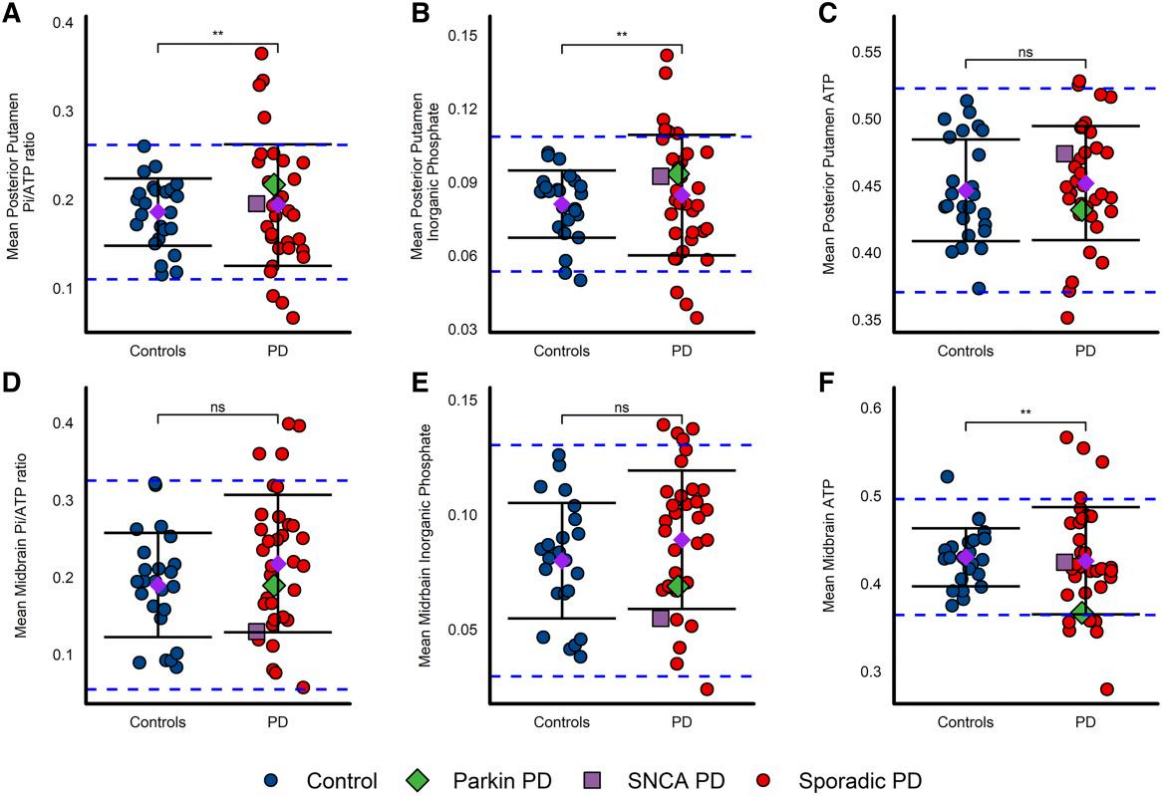
**Increased variance of putaminal, but not midbrain Pi/ATP in Parkinson's disease**. ^31^P-MRS in Parkinson's disease compared to controls. All ^31^P-MRS values are normalized to total phosphorus signal detected in the spectra. (**A**) Mean posterior putamen Pi/ATP ratio, *F*-test *P* = 0.0036; (**B**) mean posterior putamen Pi, *F*-test *P* = 0.0042; (**C**) mean posterior putamen ATP, *F*-test *P* = 0.5624. (**D**) Mean midbrain inorganic phosphate (Pi)/ATP ratio, *F*-test *P* = 0.1708; (**E**) mean midbrain Pi, *F*-test *P* = 0.3674; (**F**) mean midbrain ATP, *F*-test *P* = 0.0030. Mean (purple diamond) ± standard deviation (SD). Variances between groups were tested with the *F*-test of equality of variances and statistical significance is displayed on each panel. Blue dashed lines denote 2 SDs from the control mean values. The parkin (*PRKN*^−/−^) mutant patient is depicted with a green diamond, the *SNCA-G51D* mutant patient is depicted with a purple square. Midbrain data: controls *n* = 24, Parkinson's disease *n* = 35. Putamen data: controls *n* = 25, Parkinson's disease *n* = 35. *P < 0.05, **P < 0.01. Pi = inorganic phosphate; ns = not significant.

The variance of the Pi/ATP ratio was markedly increased in the posterior putamen in patients with Parkinson's disease compared to control subjects (Fig. 5A, *F*-test *P* = 0.0036) with 7/35 patients having Pi/ATP ratios outside 2 SDs. Nine of 35 patients had levels of Pi that fell outside 2 SDs in the posterior putamen, this included all seven participants with Pi/ATP outside 2 SDs (Fig. 5B, *F*-test *P* = 0.0042). There was no difference in variance in total ATP in the posterior putamen between groups, with only 3/35 patients being outside 2 SDs (Fig. 5C, *F*-test *P* = 0.5624). This may indicate impaired oxidative phosphorylation in the posterior putamen in those participants with particularly elevated Pi/ATP ratios. The levels of all ^31^P-MRS parameters were similar in the anterior putamen compared to the posterior putamen, although the differences in variance were less marked. This may be due to the posterior putamen being more profoundly affected in early Parkinson's disease than the anterior putamen (Supplementary Table 2).^48^

In contrast to the posterior putamen, the variance in the Pi/ATP midbrain ratio was similar in patients with Parkinson's disease and controls (Fig. 5D, *F*-test *P* = 0.1708), as were the total levels of Pi in the midbrain (Fig. 5E, *F*-test *P* = 0.3673). However, 10/34 patients still exhibited ATP midbrain values outside two control group SDs from the healthy control mean (Fig. 5F, *F*-test *P =* 0.0030). This may indicate that some participants with Parkinson's disease have altered bioenergetics in the midbrain, although this may be less marked than that of the putamen, which demonstrates clear differences in variance of the Pi/ATP ratio, which is more specific for impaired oxidative phosphorylation. The observation of elevated Pi/ATP ratios in the posterior putamen only, and not in the midbrain, supports the hypothesis of the ‘dying back’ hypothesis of nigrostriatal neurons in Parkinson's disease.^47,49^

### Higher ^31^P-MRS-derived phosphocreatine values correlate with higher risk of fast Parkinson's disease progression

We finally determined whether any of the ^31^P-MRS derived imaging parameters correlated with clinical parameters. Higher ^31^P-MRS-derived midbrain phosphocreatine correlated with a higher predicted risk of adverse outcome in disease progression, defined as the presence of either postural instability or dementia at 5 years (*r* = 0.47, adjusted *P* = 0.0384) this correlation with phosphocreatine was not present in either the posterior or anterior putamen (*r* = −0.087, adjusted *P* = 0.621 and *r* = −0.061, adjusted *P* = 0.729, respectively). There were no correlations between ^31^P-MRS-derived Pi/ATP ratio, ATP or Pi with predicted risk of adverse outcome in either the midbrain, posterior putamen or anterior putamen (data not shown). There were no correlations between ^31^P-MRS-derived Pi/ATP ratio, ATP, Pi or phosphocreatine in either the midbrain, posterior putamen or anterior putamen and both the MDS-UPDRS III score and MDS-NMSS score.

## Discussion

Many of the molecular hallmarks occurring in nigral dopaminergic neurons have also been reported in fibroblasts from patients with the sporadic or monogenic forms of Parkinson's disease.^50^ Our group had previously undertaken detailed characterization of mitochondrial dysfunction and morphology in fibroblasts from both familial and sporadic Parkinson's disease and also undertaken the first screen of an entire compound library in patient-derived tissue.^7,8,12,51^ This study is now the first to combine cellular measures of mitochondrial and lysosomal function and morphology in fibroblasts derived from patients with Parkinson's disease with ^31^P-MRS-derived quantification of bioenergetic dysfunction in the CNS *in vivo*. Multimodal, in-depth characterization of pathogenic mechanisms leading to Parkinson's disease is also likely to be particularly pertinent in early Parkinson's disease to facilitate mechanistically defined disease stratification for future clinical trials.^52^

^31^P-MRS could be considered as a potential biomarker to identify a broad phenotype of bioenergetic dysfunction in Parkinson's disease regardless of the aetiology (genetic versus sporadic) or any—as yet speculative—mechanistically defined subtypes of Parkinson's disease. ^31^P-MRS Pi/ATP ratios may therefore help to stratify participants for future neuroprotective clinical trials targeting bioenergetic dysfunction.

Phenotypes observed in fibroblasts may not correspond to functional impairments observed in neurons derived from the same patients with Parkinson's disease. However, our previous studies demonstrated consistent changes between fibroblasts and tyrosine hydroxylase-positive neurons derived from these fibroblasts for ATP, MMP and the number of lysosomes, with all changes being considerably more marked in neurons.^12^

We observed a striking increase in variance in virtually all investigated parameters in patients compared to controls. This increased variance is difficult to interpret but suggests that multiple distinct molecular mechanisms may underlie the changes observed in individual patients with Parkinson's disease. Mitochondrial function may be in compensatory ‘metabolic overdrive’ in patients with high levels of energy-rich metabolites to address increased energy demands of the cell secondary to activation of other mechanisms with high energy demands such as DNA repair or increased lysosomal activity due to protein aggregation. In contrast, reduced levels of energy-rich metabolites may suggest that mitochondrial dysfunction may play more of a central role in the pathogenesis of Parkinson's disease in these patients. We previously identified marked lowering of complex I and complex IV activity in fibroblasts of patients with ATP levels ≥ 2 SD below the mean.^12^

There are some methodological limitations that need to be acknowledged. In our fibroblast measurements, the mitochondrial and lysosomal count parameters can only be an estimation and proxy for the actual counts. There are multiple reasons for this. The fluorescent dyes used in this study, to enable multiplexing of the assay, were TMRM and LysoTracker™, both of which rely on the organelle to be somewhat functional (a membrane potential for mitochondria and an acidic pH for LysoTracker™). Although these dyes will capture most organelles, some will be missed using these live imaging fluorescence dyes. In addition, the counts are based on the high content imaging analysis, which is not an accurate measure of organelle area or volume, hence can only be an approximation of the organelle content in fibroblasts. More in-depth studies could be undertaken in a smaller proportion of cell lines, however, this would be logistically unfeasible in the whole cohort.

We identified several biologically and pathologically plausible relationships between mitochondrial and lysosomal phenotyping in peripheral tissue and putaminal ^31^P-MRS measures of bioenergetic dysfunction indicative of impaired mitophagy in Parkinson's disease.

Impaired mitophagy may be due to impairment of PINK1-dependent recruitment of parkin from the cytoplasm to damaged mitochondria in a membrane potential-dependent manner.^53^ PINK1 protein levels are decreased in both LRRK2-G2019S and sporadic Parkinson's disease fibroblasts after valinomycin-induced mitochondrial depolarization.^54^ Alternatively, downstream mechanisms may be impaired. A previous post-mortem study in Lewy body disease demonstrated an accumulation of phosphorylated ubiquitin that co-localized with markers of mitochondria and lysosomes, suggesting impaired mitophagy downstream of the initial signalling of mitochondria for degradation via ubiquitination.^10^ Our results are also in keeping with the previous observation of impaired mitophagy flux and increased mitochondrial content in a small series of sporadic Parkinson's disease fibroblast lines.^55^

Our patient cohort included two rare monogenic cases of Parkinson's disease with homozygous mutations in the parkin gene (*PRKN*) or the G51D mutation in the alpha-synuclein gene (*SNCA*), respectively. Extensive genotyping for known pathogenic mutations in other monogenic Parkinson's disease genes and any variants of GBA1 associated with increased risk of Parkinson's disease was otherwise negative. The frequency of familial cases within our cohort is therefore similar to the general observation that genetic causes of Parkinson's disease can be identified in 5–10% of cases of Parkinson's disease in a white Caucasian population.^56^ None of the results reported above changed in significance if these two familial cases were excluded (data not shown). We acknowledge that some of our controls also had a positive family history for Parkinson's disease. However, this was purely chance—controls were selected based on their age and, as far as possible, to achieve a similar gender split to the patient cohort.

Interestingly, the data for all parameters from the peripheral fibroblasts were largely the same pattern if the cells were grown in galactose or glucose-containing media. Galactose media forces the cells to rely more heavily on oxidative phosphorylation for energy generation and in cells from patients with classical mitochondrial diseases, is often used to reveal a mitochondrial-driven deficit in energy status. The results in this study suggest that the metabolic abnormalities are present in both culture conditions. Upon closer investigation of MMP specifically, the response of patient fibroblasts to galactose was more variable than in controls. In addition, patient fibroblast lines with high MMP in glucose had a reduced MMP in galactose media with a galactose:glucose ratio of <1. This could indicate that these patient fibroblasts are unable to maintain high MMP without additional metabolic pathways, revealing the mitochondrial deficit. However, little change in MMP was observed in patient fibroblasts with low MMP in glucose, suggesting less metabolic flexibility in cell lines with already impaired oxidative phosphorylation. Although longer periods of galactose treatment are possible to potentially unmask mitochondrial defects further, this can subsequently impair cell viability. We chose a 72-h period of galactose treatment to balance cell viability and exposing mitochondrial deficits whilst avoiding the confounding effect of cell death upon our assays. However, taking this into consideration, our data may reflect that there are underlying metabolic and mitochondrial deficits, which in the fibroblasts are at the baseline of what the cell type can deal with, as fibroblasts are relatively metabolically flexible and can use multiple substrates to maintain the broad mitochondrial parameters, which were measured in this study.^57,58^ However, it would be interesting and important to investigate whether this would also be the case in neurons, which are generally metabolically less flexible.

Mitochondrial uncoupling can paradoxically lead to greater concentrations of ATP and increased mitochondrial biogenesis.^46^ Mitochondrial uncoupling also induces mitophagy.^41,43^ The observed correlation of higher intracellular ATP levels with lower MMP in fibroblasts from patients with sporadic Parkinson's disease in our study combined with the indices of mitophagy discussed above, are in keeping with previous reports of increased mitophagy and mitochondrial uncoupling in LRRK2 Parkinson's disease fibroblasts and provide further support for shared mechanisms in sporadic and familial Parkinson's disease.^55,59^ We previously demonstrated that an increase in the number of lysosomes in fibroblasts of patients with Parkinson's disease closely correlates with lysosomal dysfunction, reflected by decreased cathepsin D activity.^12^ Further work is required to fully investigate mitophagy rates in these patient cells.

Mitophagy may represent a promising therapeutic target for neuroprotection.^60^ Therapies still in pre-clinical testing include inhibitors of deubiquitinating enzymes (DUBs). DUBs regulate substrate ubiquitination by E3-ubiquitin ligases such as parkin. DUB inhibitors would therefore lead to an increase in ubiquitination and increased mitophagy. So far, inhibition of DUBs has primarily been investigated as a therapeutic target for on *PRKN* or *PINK1*-related Parkinson's disease but may also be beneficial in a carefully stratified population of sporadic Parkinson's disease.^61^ Deferiprone, an iron chelator that induces mitophagy and has shown promise in many preclinical models of Parkinson's disease, is currently in a large multicentre clinical trial (FAIRPARK-II, NCT02655315).^62^

The increase in variance across many ^31^P-MRS measures of bioenergetic dysfunction is markedly similar to the increased variance for the fibroblast-derived bioenergetics measures of cellular function and likely to reflect the heterogenous aetiology of Parkinson's disease.^12^ Previous ^31^P-MRS data have been conflicting. Hattingen *et al*.^29^ reported reduced concentrations of ATP in the midbrain and reduced ATP and phosphocreatine in the putamen. Hu *et al*.^63^ identified elevated ratios of Pi/ATP in the temporoparietal region in Parkinson's disease. However, others have found no differences in Parkinson's disease compared to healthy controls in either the striatum or temporoparietal cortex.^64^ The conflicting data may represent significant methodological differences in ^31^P-MRS acquisition parameters, sample size and clinical heterogeneity, including severity and disease duration of Parkinson's disease in the patients included in these studies.

The relationships identified in Parkinson's disease between peripheral tissue and ^31^P-MRS were specific to the putamen and not identified in the midbrain. Nigral dopaminergic neurons have an exceptionally extensive axonal arbour with long, thin, unmyelinated neurons and are thought to be particularly vulnerable to impaired energy supply and cellular stressors, such as oxidative stress.^44,45,65^ There is strong evidence from post-mortem and imaging studies in Parkinson's disease that the loss of dopaminergic striatal terminals exceeds the extent of dopaminergic neuronal cell loss considerably, especially in the premotor and early phases of clinically manifest Parkinson's disease.^44,46^ Synaptic terminals have a particularly high energy demand, making them more vulnerable to mitochondrial dysfunction and oxidative stress.^66^ Recently, a rodent model of Parkinson's disease demonstrated that striatal synaptic dysfunction was preceded by early mitochondrial dysfunction and followed by autophagic decline with accumulation of autophagic vesicles and damaged mitochondria prior to loss of dopaminergic synapses and neuronal cell death.^47^ Our data are consistent with these observations and suggest that mitochondrial dysfunction and impaired mitophagy may be more prominent in the dopaminergic striatal nerve endings than the neuronal cell bodies in the midbrain. The possibility of retrograde axonal degeneration offers alternative routes to neuroprotection as targeting the early pathogenic processes that initiate axonal degeneration may ultimately prevent neuronal cell body loss, or even allow the restoration of the axonal arbour.^44,65,46^

The observed correlation between higher ^31^P-MRS-derived midbrain phosphocreatine and predicted risk of rapid disease progression (as defined by the presence of postural instability or dementia at 5 years) should be interpreted with caution and awaits confirmation in larger longitudinal studies. In the Velseboer *et al*.^15^ study, the predictive model was developed and validated in patient cohorts who were assessed at a mean of 0.3 years from diagnosis, but symptom duration at baseline was significantly longer. In the development cohort, motor symptom duration was a mean (SD) of 1.6 (0.9) years and in the validation cohort, mean motor symptom duration was 2.2 (1.8) years.^15^ Our cohort was a mean of 13.7 months from diagnosis and whilst we do not have data on symptom duration in our cohort, in other UK-based studies, the interval between motor symptom onset and diagnosis has been estimated to be ∼12 months.^67^ Hence we anticipate that Parkinson’s disease stage in our cohort is likely to be broadly comparable to the original validation cohort. The creatine-phosphocreatine system is a key high-energy buffer in the CNS and ensures an adequate supply of ATP from the breakdown of phosphocreatine to creatine and Pi by creatine kinase (CK) during metabolic activity.^68^ Elevated midbrain phosphocreatine may reflect excessive synthesis of phosphocreatine in a compensatory attempt to overcome a bioenergetic deficit, dysfunctional utilization, or both. Ubiquitous mitochondrial CK (found in all tissues) is coupled to oxidative phosphorylation, but brain-specific cytoplasmic CK is associated with glycolysis.^68^ The observation of greater risk of rapid progression in Parkinson's disease could therefore be related to changes in either mitochondrial function or glucose homeostasis.

Our study has several limitations. Correlation analyses are limited as they can only imply an association between different measurements and are not able to identify the specific biological mechanism that results in the correlation observed. However, the strengths and clear numerical differences of the correlations between Parkinson's disease and healthy controls, also observed in the figures, support a clear disease-specific relationship, as discussed above. Changes in ^31^P-MRS bioenergetic parameters, such as ATP and Pi, are not specific to oxidative phosphorylation as glycolysis also contributes to ATP production, particularly in resting protocols. Furthermore, the patient-derived tissue assessed in this study was non-neuronal, which limits direct correlations with ^31^P-MRS imaging-based results derived from brain tissue.

Nonetheless, our results suggest that a combination of these two approaches—^31^P-MRS and assessment of mitochondrial function in peripheral tissue—provide complementary perspectives and convergent evidence on bioenergetic pathways and related mechanisms in Parkinson's disease.

## Conclusion

In conclusion, we have undertaken the first multimodal assessment of mitochondrial function in Parkinson's disease, combining ^31^P-MRS quantification of energy-rich metabolites in the striatum and midbrain with in-depth characterization of mitochondrial function and morphology in peripheral tissue. Our data suggest that impaired bioenergetics in the striatal dopaminergic nerve terminals exceeds that observed in the midbrain of patients with early Parkinson's disease. Our data further support the hypothesis of a prominent link between impaired mitophagy and impaired striatal energy homeostasis as a key event in early Parkinson's disease. We propose that the combination of central and peripheral measures of mitochondrial function may be more promising for future mechanistic disease stratification than the application of a single biomarker. This would be particularly relevant to future clinical trials wishing to stratify for mitochondrial dysfunction, the rescue of which may halt retrograde axonal degeneration in Parkinson's disease and restore the synaptic arbour of nigrostriatal neurons, at least partially.

## Supplementary Material

awad364_Supplementary_Data

## Data Availability

The data that support the findings of this study are available from the corresponding author, upon reasonable request.
