# Supplementary material for: Multimodal assessment of mitochondrial function in Parkinson's disease
Source: Brain. 2023 Dec 7;147(1):267–80. doi: 10.1093/brain/awad364 (PMC10766247; doi:10.1093/brain/awad364)
Supplement: awad364_Supplementary_Data [file awad364_supplementary_data.pdf]

Supplementary information for:

Multimodal assessment of mitochondrial function in Parkinson's disease

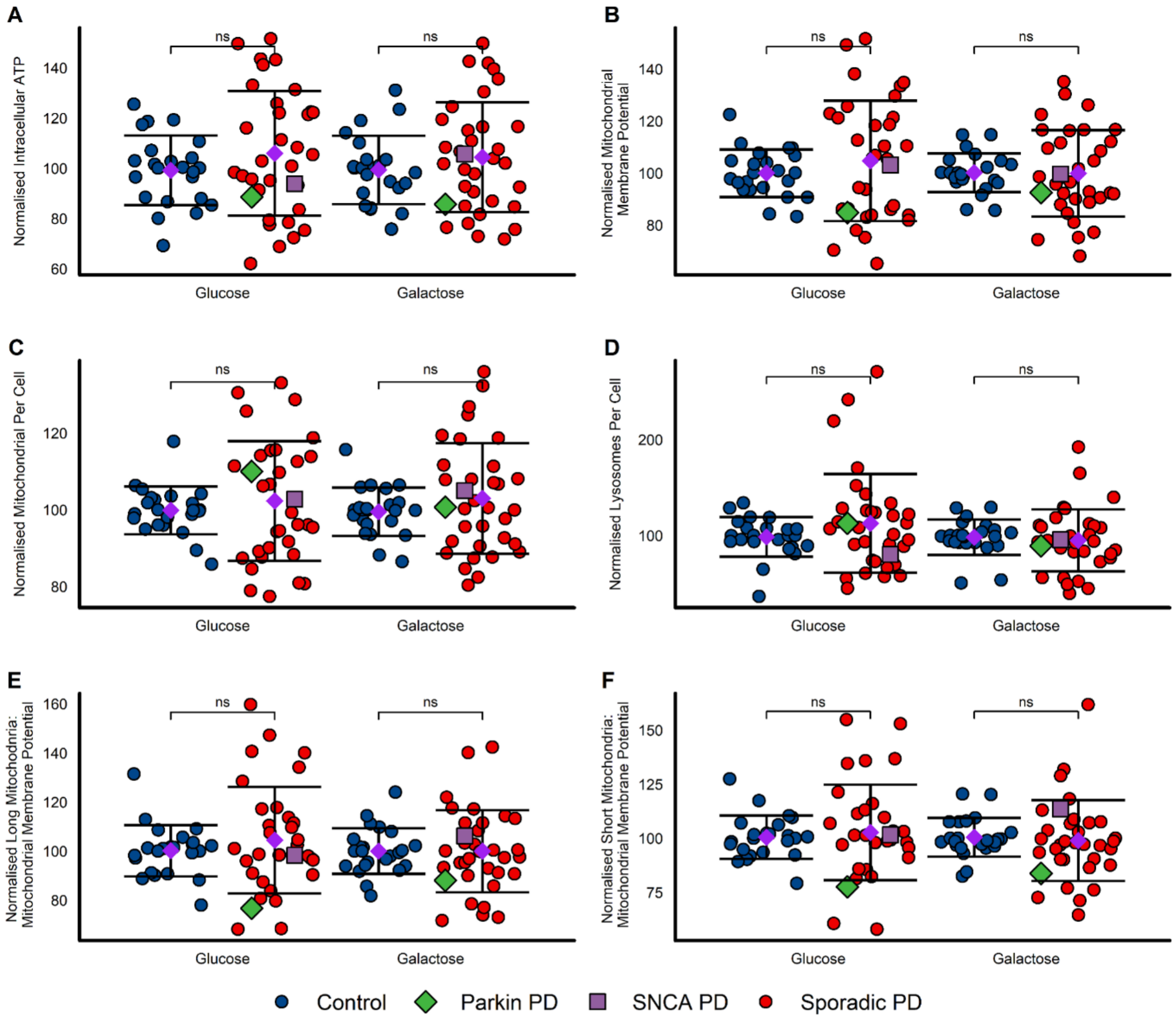

Supplementary Figure 1: Group differences in mitochondrial and lysosomal function in PD-patient derived fibroblasts across both media types. (**A**) Intracellular ATP, (**B**) MMP, (**C**) mitochondria count per cell, (**D**) lysosomal count per cell, (**E**) long mitochondria MMP only, (**F**) short mitochondria MMP. Mean (purple diamond) ± standard deviation (SD) presented. The parkin (*PRKN*$^{-/-}$) mutant patient is depicted with a green diamond, the *SNCA* G51D mutant patient is depicted with a purple square. All fibroblast assays repeated in triplicate in both glucose and galactose containing media, all values are normalised to the

control mean in glucose. Group differences tested with unpaired *t*-test with Welch's correction. ns=not significant

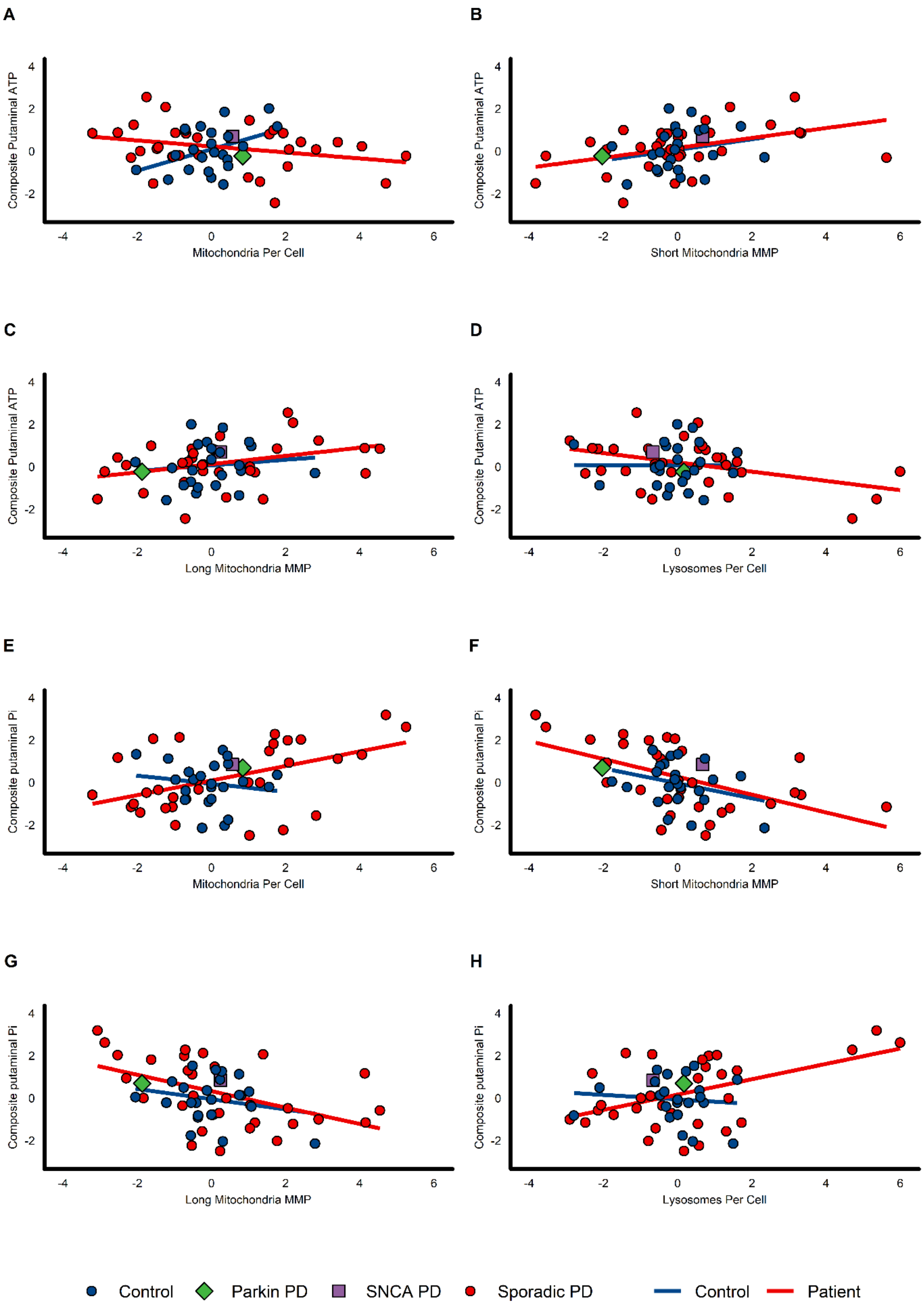
A
B
C
D
E
F
G
H
Composite Putaminal ATP
Composite putaminal Pi
Mitochondria Per Cell
Short Mitochondria MMP
Long Mitochondria MMP
Lysosomes Per Cell
4
2
0
-2
-4
6
Control
Parkin PD
SNCA PD
Sporadic PD
Control
Patient

Supplementary Figure 2: ^{31}P-MRS measured putaminal ATP and inorganic phosphate and relationships to fibroblast assay results. All ^{31}P-MRS data is expressed as composite z-scored data of all putaminal voxels. All fibroblast assay data is expressed as a composite z-score of triplicate repeats in both glucose containing and galactose containing media. Pearson's correlation coefficient was used for all analyses. The parkin (*PRKN*$^{-/-}$) mutant patient is depicted with a green diamond, the *SNCA* G51D mutant patient is depicted with a purple square. (**A**) putaminal ATP and mitochondria per cell (control *r*=0.416, *p*=0.0484; PD *r*=-0.298, *p*=0.0865), (**B**) putaminal ATP and mitochondrial membrane potential (MMP) in short mitochondria (control *r*=0.219, *p*=0.316; PD *r*=0.442, *p*=0.0088), (**C**) putaminal ATP and MMP in long mitochondria (control *r*=0.126, *p*=0.568; PD *r*=0.359, *p*=0.0372), (**D**) putaminal ATP and lysosomes per cell (control *r*=-0.003, *p*=0.991; PD *r*=-0.433, *p*=0.0105), (**E**) putaminal inorganic phosphate (Pi) and mitochondrial per cell (control *r*=-0.156, *p*=0.476; PD *r*=0.358, *p*=0.0376), (**F**) putaminal Pi and MMP in short mitochondria (control *r*=-0.307, *p*=0.155; PD *r*=-0.482, *p*=0.0039), **(G)** putaminal Pi and MMP in long mitochondria (control *r*=-0.229, *p*=0.293; PD *r*=-0.453, *p*=0.0072), (**H**) putaminal Pi and lysosomes per cell (control *r*=-0.101, *p*=0.476; PD *r*=0.358, *p*=0.0376).Putamen data; control n = 23, PD n = 34. Midbrain data; control n = 22, PD n =34.

	Control (n = 23)	Parkinson's (n = 34)		
**Intracellular ATP**	**Normalised values**	**Normalised values**	**F-test**	**T-test**
Glucose containing media	99.164 ± 13.864	105.928 ± 24.845	**	ns[a]
Galactose containing media	99.317 ± 13.649	104.386 ± 21.872	*	ns[a]
**Overall Mitochondrial Membrane Potential**				
Glucose containing media	99.902 ± 9.114	104.64 ± 23.127	***	ns[a]
Galactose containing media	100.096 ± 7.420	99.858 ± 16.621	***	ns[a]
**Short Mitochondria Mitochondrial Membrane Potential[a]**				
Glucose containing media	100.458 ± 10.019	102.678 ± 22.102	***	ns[b]
Galactose containing media	100.420 ± 8.984	98.896 ± 18.679	***	ns[b]
**Long Mitochondria Mitochondrial Membrane Potential [a]**				
Glucose containing media	100.108 ± 10.438	104.444 ± 21.702	***	ns[b]
Galactose containing media	99.989 ± 9.317	99.955 ± 16.724	**	ns[b]
**Mitochondria Per Cell**				
Glucose containing media	99.810 ± 6.236	102.268 ± 15.571	***	ns[a]
Galactose containing media	99.471 ± 6.287	102.954 ± 14.934	***	ns[a]
**Lysosomes per cell[a]**				
Glucose containing media	98.505 ± 20.522	112.592 ± 51.366	***	ns[b]
Galactose containing media	98.109 ± 18.427	94.895 ± 32.303	**	ns[b]

Supplementary Table 1: Fibroblast assay summary results. Mean and standard deviation and f-test shown for all fibroblast data in both glucose and galactose containing media. Group differences in fibroblast parameters assessed with t-tests. [a]Independent samples t-test with Welch's correction. [b]Group differences tested with Mann-Whitney U test as data was non-normally distributed. ns=not significant, *<0.05, **<0.01, ***<0.001.

^{31}P-MRS Parameters	Control (n = 24)	Parkinson's (n = 35)		
**Total ATP**	**Amplitude**	**Amplitude**	**F-test**	**β_{group}**
Mean midbrain	0.430 ± 0.033	0.426 ± 0.061	**	ns[a]
Mean posterior putamen	0.446 ± 0.038	0.452 ± 0.043	ns	ns[a]
Mean anterior putamen	0.430 ± 0.043	0.439 ± 0.040	ns	ns[a]
**Total inorganic phosphate**				
Mean midbrain	0.080 ± 0.025	0.089 ± 0.030	ns	ns[a]
Mean posterior putamen	0.081 ± 0.014	0.085 ± 0.025	**	ns[a]
Mean anterior putamen	0.082 ± 0.016	0.088 ± 0.026	*	ns[a]
**Inorganic phosphate/ATP ratio**				
Mean midbrain	0.190 ± 0.068	0.217 ± 0.089	ns	ns[a]
Mean posterior putamen	0.186 ± 0.038	0.194 ± 0.069	**	ns[a]
Mean anterior putamen	0.197 ± 0.049	0.206 ± 0.073	ns	ns[a]
**Total phosphocreatine**				
Mean Midbrain	0.192 ± 0.021	0.186 ± 0.028	ns	ns[a]
Mean posterior putamen	0.195 ± 0.018	0.197 ± 0.021	ns	ns[a]
Mean anterior putamen	0.202 ± 0.017	0.203 ± 0.022	ns	ns[a]
**Total phosphorus signal detected**				
Mean midbrain	4.959 ± 0.970	4.911 ± 0.928	ns	ns[b]
Mean posterior putamen	7.106 ± 0.746	6.970 ± 1.224	ns	ns[b]
Mean anterior putamen	6.981 ± 0.575	6.745 ± 1.007	ns	ns[b]

Supplementary Table 2: ^{31}P-MRS summary results. Mean and standard deviation and f-test shown. All ^{31}P-MRS amplitudes are normalised to the total phosphorus signal detected within the respective voxel. Of note there was no difference in total phosphorus signal between groups in any voxel examined, therefore demonstrating justification for normalising to total phosphorus signal. [a]Group differences in ^{31}P-MRS parameters were tested with linear regression controlling for age and sex, the significance of the β coefficient for group effect is reported (β_{group}). [b]Group differences in total phosphorus signal were assessed with unpaired t-tests. ns=not significant, $**p<0.01$,
